# Challenges in Assessing Blood Pressure in Adults Following Intravenous Paracetamol Infusion in the Emergency Room

**DOI:** 10.7759/cureus.43355

**Published:** 2023-08-11

**Authors:** Fatima M Alameri, Khalid Alkhalaileh, Yousif Alkhafaji, Thiagarajan Jaiganesh, Satish C Nair

**Affiliations:** 1 Department of Emergency Medicine, Tawam Hospital, Al Ain, ARE; 2 Medical Research, Tawam Hospital, Al Ain, ARE

**Keywords:** blood pressure, pain, united arab emirates, gulf, middle east

## Abstract

Background and objective

The negligible side effects of paracetamol along with its ease of availability have catapulted paracetamol to be a widely used medication in emergency room management to reduce pain and subsequent elevations in blood pressure (BP). Our study aimed to address the challenges in informed clinical decision-making in the emergency room following paracetamol intravenous infusion.

Methods

This was a retrospective cross-sectional study involving the extraction of data from electronic medical records of patients who received intravenous paracetamol infusion between January 2022 and May 2022. Demographic information and BP-related data were collected for analysis.

Results

We initially considered a total of 162 patient records, with 132 of them eventually meeting the inclusion criteria. Among patients receiving paracetamol infusion for 15 minutes or less, 34% showed a drop of 1-5 mmHg in systolic BP (SBP), while 26% experienced a drop of 6-10 mmHg. However, infusion time longer than 16 minutes did not significantly reduce SBP. Diastolic BP (DBP) was not significantly affected by the duration of the paracetamol infusion. Analysis of the drop in SBP revealed no significant associations with age, gender, or ethnicity. Mean arterial pressure (MAP) was not significantly affected by the duration of paracetamol infusion.

Conclusion

Our findings suggest that intravenous paracetamol infusion does not significantly lower BP in adults in the emergency room, except for infusions of shorter durations. However, various factors, including infusion rate, patient characteristics, and concomitant medications, may influence BP measurements. The study emphasizes the need for establishing standardized criteria and conducting further research to assess intravenous paracetamol's hemodynamic effects accurately.

## Introduction

Acute pain produces a stress response that causes a transient increase in blood pressure (BP) in adults [1]. In contrast, chronic pain, through the impaired regulation of the cardiovascular, and analgesic systems, may cause a long-lasting increase in BP [2]. Several analgesics have been reported to have BP-related effects, which vary according to the type of drug used. The non-opioid drug paracetamol works by blocking central cyclooxygenase (COX) and activating serotoninergic pathways in the spinal cord to alter the central nervous system (CNS) [3]. The injectable formulation of the drug contains 10 mg of effective ingredient per ml and should be injected intravenously. Paracetamol is a common over-the-counter analgesic used to ameliorate mild to moderate pain, the symptoms of colds and flu, and reduce fever. To treat pain and prevent further BP elevations, paracetamol is frequently utilized in emergency rooms because of its minimal risk of adverse effects and ease of accessibility [4-5]. However, little is known about the hemodynamic effects of intravenously administered paracetamol. The purpose of this study is to ascertain whether intravenous paracetamol infusion impacts hemodynamic measures, especially BP, and the challenges associated with informed clinical decision-making in the emergency department.

## Materials and methods

Study design

This was a retrospective single-center cross-sectional study performed in the Emergency Department of a tertiary academic medical center in Al Ain, the Eastern Region of the Emirate of Abu Dhabi in the UAE [6]. The extraction of data from patient electronic medical records was conducted by volunteer medical resident researchers blinded to the study hypothesis. It was hypothesized that intravenous paracetamol infusion impacts hemodynamic measures, especially BP in adults (alternative hypothesis). On the other hand, the idea that intravenous paracetamol infusion does not impact hemodynamic measures such as BP in adult patients served as the null hypothesis. Electronic patient medical records were accessed to gather data on patients treated between January 2022 and May 2022.

Patient information 

The medical charts of all patients receiving intravenous paracetamol during the period from January 1, 2022, to May 31, 2022, in the emergency room were analyzed for the study. Patient charts of patients receiving intravenous paracetamol infusion were accessed, and the patients were classified into two groups based on the infusion duration of paracetamol. Group 1 consisted of 50 patients who received intravenous paracetamol for 15 minutes or less, and Group 2 consisted of 82 patients who received intravenous paracetamol for >16 minutes. In fact, the stringent inclusion selection criteria for patients receiving IV paracetamol for 15 minutes or less reduced the numbers in this group. Demographic details data on systolic BP (SBP) and diastolic BP (DBP) were harvested from patient medical records using the International Classification of Diseases, Tenth Revision-Clinical Modification (ICD10-CM) codes.

Inclusion/exclusion criteria

The inclusion criteria were as follows: patients who were ≥18 years old and seen in the ED; chief complaint of acute pain corresponding with a visual analog scale (VAS) score ≥40 mm. Patient charts were excluded if they had any medical history (dementia, cognitive impairment) that would impair accurate pain assessment (as per the clinician’s discretion) or in cases of chronic pain, known or suspected opiate dependence, allergy to paracetamol, any degree of renal or hepatic insufficiency, pregnant patients, or those who had taken paracetamol within the last six hours before presentation.

Ethical consideration

Patient privacy and data confidentiality were ensured by adhering to the International Conference on Harmonization Good Clinical Practice (ICH-GCP) and local Department of Health Abu Dhabi guidelines. The study was approved (CRD623) by the Tawam Hospital Human Research Ethics Committee (lic#02/2009).

Data analysis

Results were presented as mean ± standard deviation (SD) for quantitative variables and were summarized as frequencies (percentages) for categorical variables. Analysis of variance was conducted to determine the statistical differences between the groups. Data were analyzed using IBM SPSS Statistics version 23.0 (IBM Corp., Armonk, NY), and p-values less than 0.05 were considered statistically significant.

## Results

The demographics of the participants are shown in Table 1. Data were initially gathered from a total of 162 patient medical records, but only 132 charts that fit the inclusion criteria were used for the study. The number of male and female participants was almost equal (Table 1). The majority of the participants were from the Middle East, including the Gulf Cooperation Council (GCC) countries (65/132, 49.2%) compared to Asia and the rest of the world. More than 60% (66%) of the participants were in the age group of 26-60 years (Table 1).

**Table 1:** Demographics of patients included in the study

Variable	N	%	Total (N)
Gender			
Male	65	49.2	132
Female	67	50.8	
Ethnicity			
Middle Eastern	65	49.2	132
Asian	47	35.6	
Others	20	15.2	
Age group, years			
18-25	19	14.4	132
26-40	44	33.3	
41-60	42	31.8	
61+	27	20.5	

Of note, 34% (17/50) of the patients experienced a 1-5 mmHg drop in SBP (Table 2), when the intravenous paracetamol infusion time was equal to or less than 15 minutes (Table 2). Similarly, 26% (13/50) of the patients showed a drop of 6-10 mmHg (p<0.05) (Table 2). In contrast, infusion time greater than 16 minutes did not significantly cause a drop in the patient’s SBP: only 23% of patients (17/82) had a drop of SBP by 1-5 mmHg, while 20% of patients (17/82) experienced a drop of 6-10 mmHg (Table 2). Paracetamol infusion duration of either 15 minutes or less or 16+ minutes did not lead to a drop (0 mmHg) in SBP in 20/50 (40%) and 46/82 (56%) patients (Table 2). None of the patients in these groups experienced a drop in SBP of more than 10 mmHg. The mean drops in SBP were 6.5 mmHg and 4.36 mmHg for the infusion times of <15 minutes and >16 minutes, respectively.

**Table 2:** Systolic blood pressure drop in patients following IV paracetamol infusion *Statistically significant

Infusion duration, minutes	Systolic blood pressure drop				
	0 mmHg	1-5 mmHg	6-10 mmHg	Total	P-value
	N (%)	N (%)	N (%)	N	
16+	46 (56.0)	19 (23.2)	17 (20.7)	82	
≤15	20 (40)	17 (34)	13 (26)	50	<0.05*

Paracetamol infusion failed to significantly lower DBP in the patients, regardless of whether the infusion time was 15 minutes or less or 16+ minutes (Table 3).

**Table 3:** Diastolic blood pressure in patients following intravenous paracetamol infusion

Infusion duration, minutes	Diastolic blood pressure drop				
	0 mmHg	1-5 mmHg	6-10 mmHg	Total	P-value
	N (%)	N (%)	N (%)	N	
16+	40 (48.7)	23 (28.1)	19 (23.1)	82	
≤15	24 (48)	14 (28)	12 (24)	50	<0.61

The drop in the SBP in patients with paracetamol infusion for 15 minutes or less was further analyzed to assess if age, gender, or ethnicity were associated with the outcomes. Thirty patients, irrespective of their ages (30/50), infused for 15 minutes or less showed a drop in their SBP by 1-10 mmHg (Table 4).

**Table 4:** Association between age and systolic blood pressure drop

	Age group, years	Systolic drop			Overall	Total patients
		0 mmHg, n	1-5 mmHg, n	6-10 mmHg. n	N	
	18-25	9	1	1	2	11
	26-40	3	11	8	19	22
	41-60	3	4	2	6	9
	61+	5	1	2	3	8
Total		20	17	13	30	50

More than 60% (19/30, 63%) of the total patients who experienced a drop in SBP were in the age group of 26-40 years (p<0.05), which was very high when compared to the other age groups: 41-60 years: 6/30, 20%; 61+ years: 3/30, 10%; and 8-25 years: 2/30, 6.6%. Ethnicity and gender were not associated with the outcomes of paracetamol infusion for 15 minutes or less in terms of inducing lower SBP. Paracetamol infusion did not significantly lower the mean arterial pressure (MAP) in the patients, regardless of whether the infusion time was 15 minutes or less or 16+ minutes.

## Discussion

Intravenous paracetamol is shown to be iso-osmotic with a pH value range of 5.0-7.0, which is similar to plasma values and unlikely to increase pain [7]. Reports indicate that in pediatric patients, infusions of mannitol and normal saline did not result in any decrease in baseline BP [7-8]. The same study successfully demonstrated that pediatric patients below the age of 18 years tolerated rapid paracetamol infusions well [7-8]. Paracetamol was infused effectively within a median time of three minutes without causing pain or showing any objective signs of local inflammatory reactions, which is consistent with previous findings among adults [7].

Our study aimed to assess the hemodynamic effect of intravenous paracetamol infusion in adults in the emergency room of a tertiary care hospital. Paracetamol infusion of less than 15 minutes caused a significant drop in SBP, but not DBP. Approximately 30/50 (60%) of the total patients receiving intravenous infusions for less than or equal to 15 minutes showed a drop in SBP (Table 2). Despite the drop in SBP observed, significant changes in the DBP and MAP were not observed in our study (Table 3). This can be attributed to the fact that arterial pressure varies over time and diastole lasts longer than systole, and MAP, the weighted average, gives more weight to diastolic pressure. Other reports have suggested that intravenous paracetamol infusion can contribute to reduced BP up to 120 minutes, post-infusion [9].

The lowering of BP following intravenous paracetamol infusion may be caused by a reduction of both cardiac output and systemic vascular resistance. Notably, the formulations of intravenous paracetamol contain mannitol as a stabilizing compound. It is likely that mannitol contained in the intravenous paracetamol formulations may have contributed to the diuretic effect to reduce BP [9]. Among the patients experiencing a drop in SBP, 63% (19/30) were between the ages of 26-41 years (Table 4), which is in line with the findings of others [4]. The exact cause for the variability and the reason behind the fact that a sizable percentage of patients did not experience a significant reduction in BP post-infusion in our study remain unknown. Several factors can be attributed to the variances observed. Firstly, the recommended infusion duration of 15 minutes is based on the experience with the treatment of infusion-related pain by propacetamol and lacks theoretical or clinical evidence to support it. Secondly, the study included a mixed pool of patients with different medical conditions, both critical and non-critical. Hence, it is important to stratify patient groups based on their severity of illness, e.g., based on the APACHE scores [10]. Thirdly, concomitant fluids and medications given alongside the paracetamol infusions also affect BP measurements [9]. Finally, different nursing staff members recorded the infusion start and stop time, thereby leading to potential variations in measurements.

Our study has several limitations, which are as follows: (a) preexisting data recorded in the electronic medical records were extracted for this study, and hence selection bias and operator bias may have crept in; (b) the results are representative of the period of the study and may not be generalizable; (c) temporal relationships between the paracetamol infusion and a drop in BP may compound the results; (d) the total effective patient population data gathered were relatively low: only 132 records for the period of study; (e) patient assessment information such as the chief complaint, pain score, and illness score were not available for the study; and (f) retrospective chart reviews are inferior in terms of the strength of the evidence, and can show association but not causation. However, this is perhaps the first study of its kind from the Persian Gulf region, and the emergency department of the hospital where the study was conducted caters to the population of the entire Eastern Region, adding to the strengths of this study.

## Conclusions

Although our study indicates that the rapid infusion (less than 15 minutes) of paracetamol to manage pain is optimal, it still continues to be a clinical challenge, especially when hemodynamic parameters such as BP changes are determined. Paracetamol infusion and the variability associated with the assessment of BP changes must be carefully addressed by taking into account patient characteristics, the severity of illness, the concomitant fluid administered, and the infusion time, in order to make informed clinical decisions.
